# Digital Leadership in Education: A Bibliometric Analysis of Research Trends from 2010 to 2024

**DOI:** 10.12688/f1000research.166667.1

**Published:** 2025-07-11

**Authors:** John Olayemi OKunlola, Suraiya Rathankoomar Naicker

**Affiliations:** 1 Department of Education Leadership and Management, Faculty of Education, P.O.BOX 524, University of Johannesburg, Auckland Park, Gauteng, 2006, South Africa

**Keywords:** digital leadership, educational technology, bibliometric analysis, AI in education, leadership 4.0, thematic evolution, collaboration network

## Abstract

The Fourth Industrial Revolution (4IR), the COVID-19 pandemic, and the rapidly digitising educational system due to advances in artificial intelligence (AI) have made a change in leadership imperative. A key framework for improving organisational effectiveness in handling these changes is digital leadership. It combines technological competencies with traditional leadership. Even with increased scholarly interest, there remains a gap in the thorough analysis of the field’s intellectual framework, thematic evolution, and collaborative dynamics. This study addresses this gap by conducting a bibliometric analysis of digital leadership research in education from 2010 to 2024, employing RStudio to map publication trends, influential sources, author productivity, conceptual themes, and social structures. Data from 338 Scopus-indexed documents reveal a significant rise in publications post-2010, peaking in 2023, with core journals such as Education and Information Technologies and Cogent Education dominating the field. Prolific authors like Karakose T., Altinay F., and Z. underscore the centrality of collaborative research, while thematic mapping identifies key clusters: digital competence, virtual leadership, and institutional innovation. Thematic evolution highlights a post-pandemic pivot toward digital transformation and AI integration, though niche areas like K-12 digital leadership remain underdeveloped. Social network analysis reveals fragmented yet growing global collaborations, with the United States, Turkey, and the United Kingdom as dominant hubs, while disparities persist in Global South participation. The study’s implications emphasize the need for interdisciplinary research, equitable global partnerships, and policy frameworks that prioritize digital leadership training for educators. Practitioners are encouraged to implement adaptive strategies to leverage emerging technologies, ensuring sustainable learning outcomes. This analysis provides a foundation for future research and practice in digital leadership by delineating the field’s conceptual and social networks, thereby bridging the divides between theory, policy, and institutional practice.

## Introduction

The Fourth Industrial Revolution (4IR) is characterised by its transformative impact on sectors including health, education, the economy, engineering, and defence through digitisation, significantly improving human life and the environment. It appears to have surpassed other successive industrial revolutions (IR) in adding value and virtues to the globe ( Okunlola et al., 2024). Hence, digitalization is profoundly revolutionizing various aspects of everyday life across sectors and industries, requiring leaders to integrate digital competencies into traditional leadership approaches for organizational feat ( Karakose et al., 2022; Oberer, B. and Erkollar, 2018). Due to this paradigm shift triggered by technological advancements, digital technologies have quickly transformed how societies function; hence, digital leadership has emerged as a new necessity for organizations to operate effectively ( Karakose et al., 2024; Okunlola, 2024a, 2024b). The wave of digitalization seems pervasive and is changing all workplaces into digital ones ( Arham et al., 2023; Ghavifekr & Pei, 2023; Karakose et al., 2023). As a result, research on digital leadership has drawn interest from scholars around the globe, and numerous studies have been carried out on the subject ( Hamzah et al., 2021; Karakose et al., 2022).

The nexus of information technology and human resources has given rise to digital leadership, which focuses on how both technical and human factors impact corporate digital transformation and supports an organization’s socio-technical aspects ( Gierlich-Joas et al., 2020; Yulianto et al., 2023). Digital leadership encompasses a variety of elements, including relationships, technology leadership, virtual teams, globalization, learning, e-leadership, leadership 4.0, and organizational performance. The umbrella term describes the role and abilities of digital leaders in the digital age ( Karakose et al., 2022; Yulianto et al., 2023). Research on digital leadership roles has increased as a result of the substantial impact that digital trends have on educational institutions. Digital leadership requires models centered on technology that support school operations ( Zhong, 2017). Education is changing due to digital technologies, and these transformative trends have necessitated the re-evaluation of leadership paradigms to effectively navigate the complexities of the digital era ( Cabellon & Ahlquist, 2016; Connolly et al., 2023). The advances in Artificial Intelligence (AI) and COVID-19 have also led to a surge in research on digital leadership in education, underscoring the need for more studies on digitalization in education ( Karakose et al., 2024).

The research on digital leadership in education is expanding and growing in leaps and bounds. Hence, this study employs a bibliometric analysis due to its capacity for science mapping and visualization in processing and analyzing large volumes of scientific data. Bibliometric analysis has now become an essential component of research evaluation methodology, which enables the unpacking of evolutionary and emerging trends in several fields of research domains ( Donthu et al., 2021; Ellegaard & Wallin, 2015; Patra et al., 2006; Smyrnova-Trybulska et al., 2017). Thus, this study offers researchers and practitioners many benefits, including illuminating the digital leadership field’s evolving development and highlighting new areas of study within it ( Donthu et al., 2021; Tigre et al., 2023). This perceived gap in understanding how the field has conceptually evolved necessitates using the bibliometric method. This study, therefore, aims to address this gap by conducting a bibliometric analysis of digital leadership research in education published between 2010 and 2024. Through the mapping of the field’s conceptual structure and thematic evolution, this research provides a deeper understanding of the dominant themes, their development, and areas that may require further investigation to guide the effective practice of digital leadership in educational institutions. Hence, the study is guided by the following research questions:

1. What is the trend in the number of publications on digital leadership in education from 2010 to 2024?
2. What are the sources of publications on digital leadership in education from 2010 to 2024?
3. What is the information about the authors with publications from 2010 to 2024?
4. What are the most common themes, keywords, and concepts on digital leadership in education from 2010 to 2024?
5. What is the social structure of the publications on digital leadership in education from 2010 to 2024?

## Literature review

### Digital leadership in education

Digital leadership in education entails adopting and utilizing new technologies to change the roles and responsibilities of principals and turn schools into digital learning environments ( Flanagan & Jacobsen, 2003; Karakose et al., 2024; Zhong, 2017). A key component of learning and leading in the pandemic and post-pandemic era appears to be incorporating digital technologies into the educational landscape ( Okunlola, 2024a). Hence, as the world changes in the post-pandemic era, school leaders need to remain current on digital transformations by keeping up with the emerging tools of the digital revolution to foster growth and development in the educational ecosystem ( Okunlola et al., 2024). However, Sheninger (2014) points out that some school administrators lack digital technology competencies and are reluctant to embrace digital technologies. Meanwhile, integrating digital leadership into education is no longer negotiable in the current age, as studies have affirmed a correlation between principals’ knowledge of technology integration and motivation to lead school-wide changes ( Hamzah et al., 2021; Leong et al., 2016; Okunlola, 2024a). The position of Van Niekerk and Van Wyk (2014) is highly instructive, and the principal is a pivotal player in bringing technology into the classroom through daily management and administrative processes.

Moreso, a digital education leader, as Sheninger opined, must demonstrate some attributes, including proficiency in communication, public relations, branding, student engagement, professional development, and innovative learning environments. Nugroho (2015) also contends that digital leadership in education requires access to high-quality resources and digital technology, and teachers who are proficient in using it ( Ridho et al., 2023). In addition, digital leadership in education places a strong emphasis on using technology effectively, reflecting critically on it, and taking steps to address how it affects school transformation ( Brown et al., 2016; Ridho et al., 2023; Rusnati & Gaffar, 2020). The current wave of digital transformations has shown that the pandemic imposed unique obligations on digital leaders. The post-pandemic era has thrust extra digital roles and responsibilities on school leaders. Leaders may follow the trend or fall behind ( Okunlola, 2024a). In other words, school leaders and teachers can no longer ignore a defining factor of current students’ exposure to technology. This makes it more imperative to adopt and apply digital leadership in education in the learning environment.

## Materials and methods

The current study employs a bibliometric methodology with the application of RStudio software (R version 4.5.0, 2025-04-11 ucrt) ( Aria & Cuccurullo, 2017). A bibliometric study is a methodical, objective, and quantitative way to map the research field and examine the bibliographic material ( Tigre et al., 2023; Zupic & Čater, 2015). Thus, a thorough grasp of the evolution of digital leadership in education is possible through bibliometric tools and network analysis, which analyze unstructured data and make significant contributions to the field ( Donthu et al., 2021). The bibliometric analysis was used to map the research growth and development of digital leadership in education from 2010 to 2024. The software can map and analyze research trends, cited references, and cited documents, publication sources, authors’ information, and conceptual, social, and intellectual structures.

### Data search strategy, extraction, and analysis procedure

**Figure 1.**
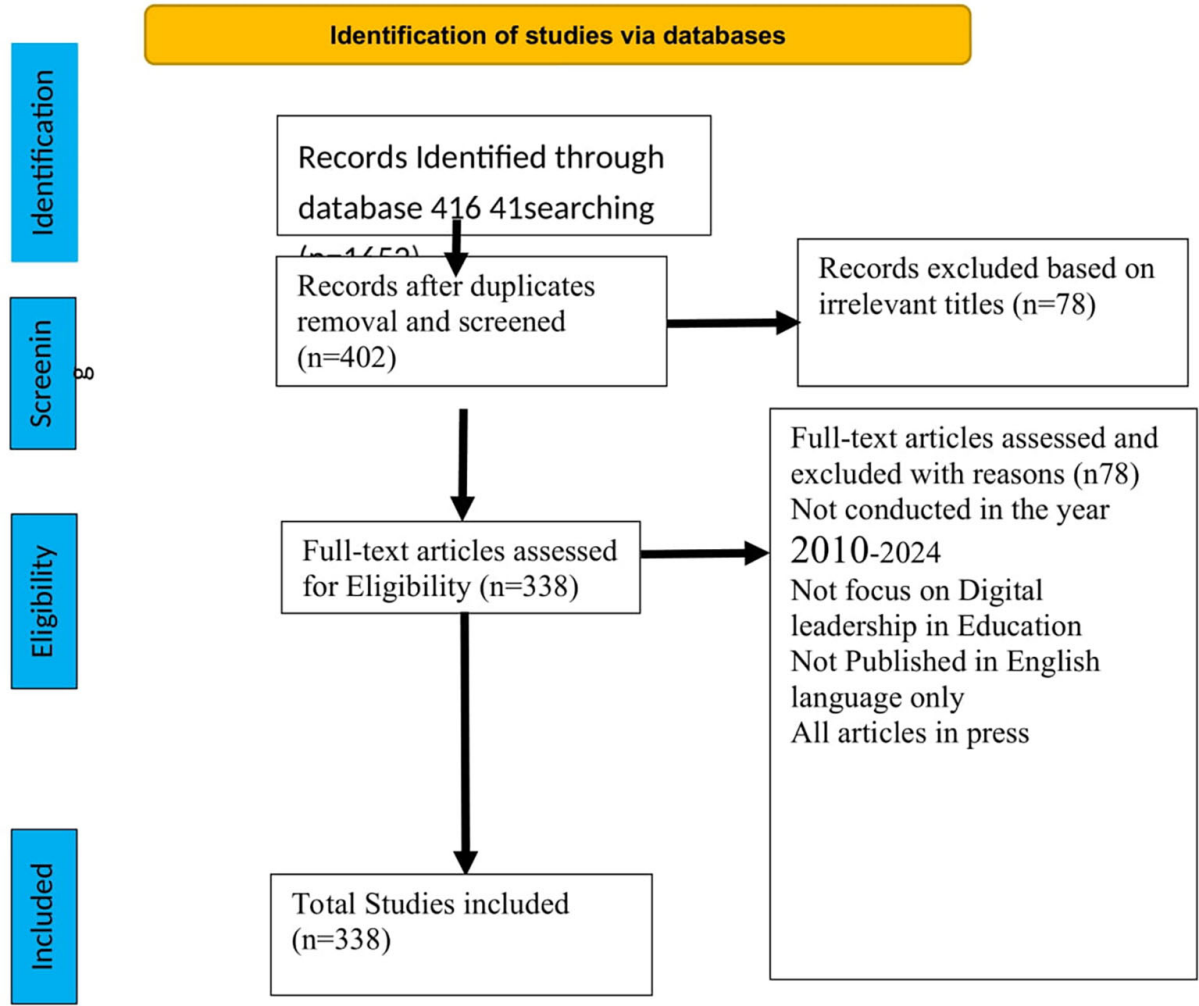
Adapted PRISMA framework for study selection.

The metadata was conducted on the Scopus database to identify and map relevant publications on digital leadership in education. Scopus is often preferred in bibliometric studies due to its broader journal coverage, reducing the risk of missing relevant articles compared to Web of Science and other databases ( Karakose et al., 2022; Mongeon & Paul-Hus, 2016). We employed search terms such as “digital leadership*” OR “schools” OR “education” OR “institution” OR “college” and 416 documents appeared in the Scopus database. The inclusion criteria benchmark requires that the document be a journal article, conference paper, book chapter, or review article published in English and at the final publication stage from 2010 to 2024. All articles in the press and not written in English were excluded, and the remaining publications that met this standard threshold were 338 documents. The document types comprise 209 journal articles, 63 conference papers, 49 book chapters, and 17 review papers. Consequently, the data analysis was conducted using the RStudio software (R version 4.5.0, 2025-04-11). Figure 1 below presents a flowchart of data search and extraction procedures based on the Preferred Reporting Items for Systematic Reviews and Meta-Analyses (PRISMA) ( Liberati et al., 2009).

## Results

### Bibliometric analysis

The bibliometric analysis revealed 338 documents published between 1993 and 2024, sourced from 249 journals, books, and other outlets. These documents have accumulated 13,724 references and exhibit an average citation count of 8.932 per document, with an annual growth rate of 10.72% and an average age of 4.84 years. The document types comprise 209 journal articles, 63 conference papers, 49 book chapters, and 17 review papers. The keyword distribution includes 1,198 Keywords Plus and 1,133 Author’s Keywords. In terms of authorship, the dataset involves 986 authors, with 73 single-authored documents and an average of 3.07 co-authors per document, of which 16.27% involve international collaboration.

**Figure 2.**
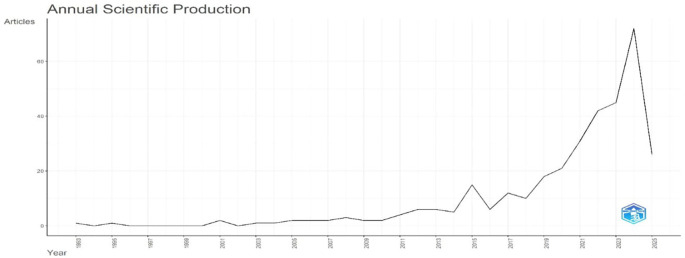
Annual scientific production. Source: Processed data outcomes (2025).

**Figure 3.**
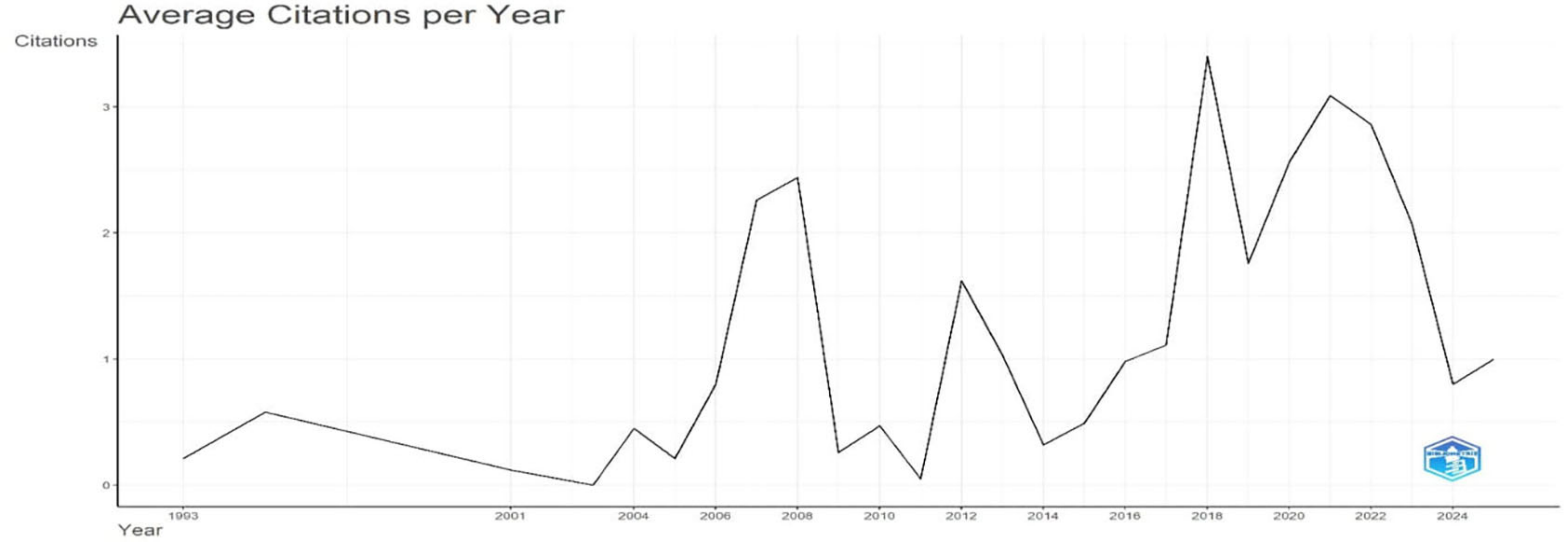
Average citations per year. Source: Processed data outcomes (2025).

Research question one explores publication trends in digital leadership in education from 2010 to 2024. Figure 2 shows a sharp post-2010 rise in publications, peaking in 2023. Figure 2 presents citation growth, mirroring publication trends, and Figure 3 maps influential leadership authors and their citation links to digital education themes.

**Research question 1**: What is the trend in the number of publications on digital leadership in education from 2010 to 2024?

According to the number of articles published annually, Figure 2 shows the evolution of research output from 1993 to 2025. The trend indicates a comparatively stagnant period in the preceding years, especially from 1993 to 2009, when the annual output was continuously low and showed little fluctuation. Around 2010, there is a noticeable upward trend that reflects a steady rise in scholarly activity. After 2017, this growth picks up speed as it enters an exponential expansion phase. The highest number of articles published during the observed period, 2023, marks the peak in scientific output. However, the notable decline observed in 2024 indicates that the trend experiences a steep drop immediately afterward. The graph highlights a notable evolution in research production overall, with consistent growth, a peak of high productivity, and a discernible recent decline.

Figure 3 shows the average number of citations per year for academic publications from 1993 to 2025. According to the data, the citation rate was comparatively constant in the early years before gradually rising beginning in 2010. After 2017, this upward trend becomes more noticeable, indicating that scholarly works are more visible and influential, perhaps as a result of more collaborative research projects and easier access to digital resources. The 2023 average citation peak indicates the highest scholarly impact during the study period. However, an apparent decrease in 2024 might be explained by matters like publication saturation, changes in the focus of research, or outside disturbances that impact citation patterns. Overall, the figure highlights times of growth and decline in citation patterns, underscoring the dynamic character of academic influence over time.

**Figure 4.**
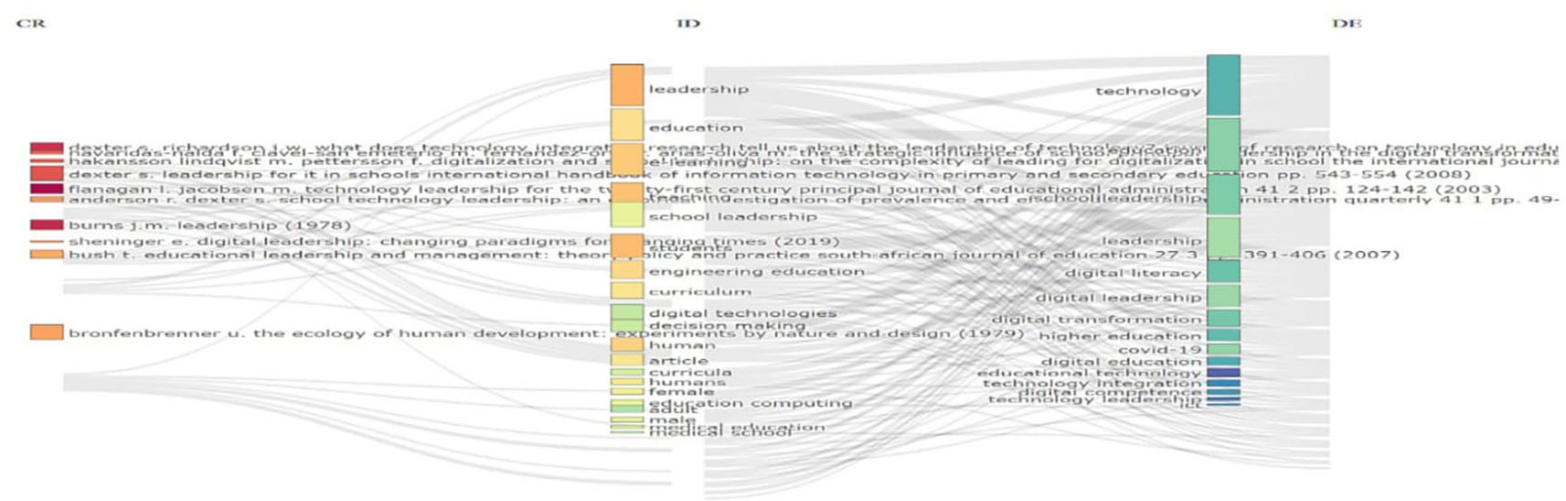
Sankey citation flow map. Source: Processed data outcomes (2025).

Figure 4 shows a citation network visualization that maps relationships between leadership and technology in education literature. Four source authors (Hector, Burns, Sheppard, Bronfenbrenner) on the left connect to various educational technology themes on the right via gray citation lines. Key themes include technology leadership, digital leadership, digital literacy, digital transformation, and educational computing. The diagram illustrates how foundational leadership theories have influenced educational technology applications, revealing the interdisciplinary connections that form the intellectual basis of technology leadership in education. The network structure demonstrates which authors have been most influential across multiple domains in this field.

**Figure 5.**
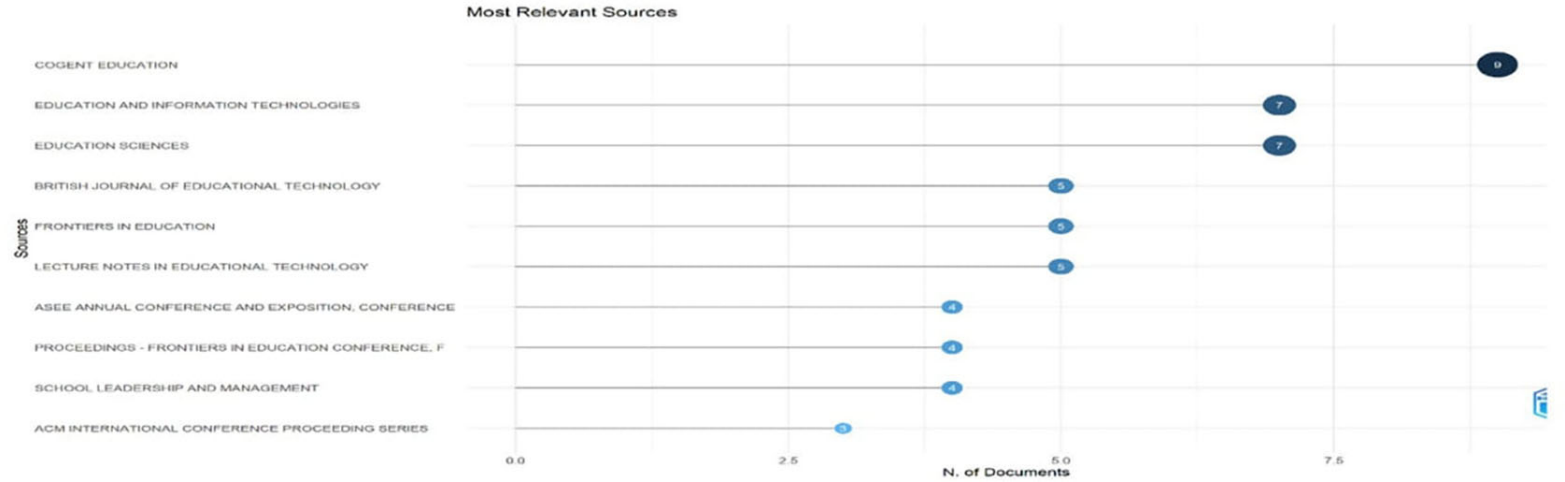
Most relevant sources. Source: Processed data outcomes (2025).

**Figure 6.**
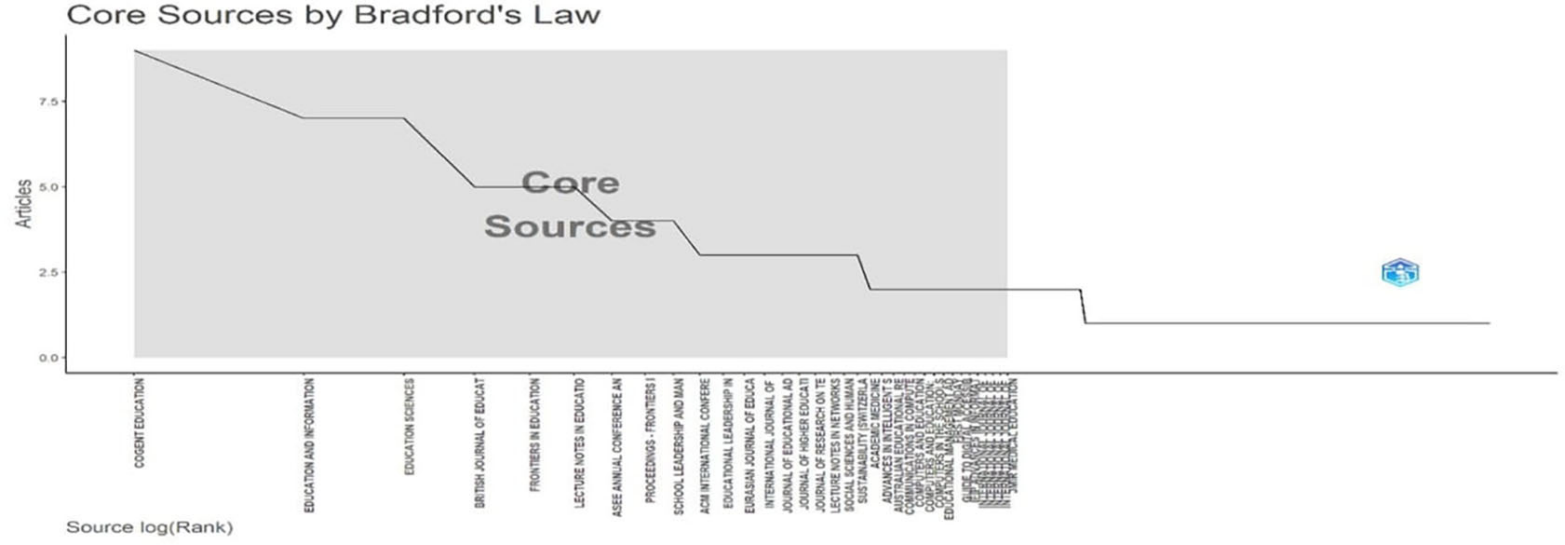
Core sources by Bradford’s law. Source: Processed data outcomes (2025).

**Figure 7.**
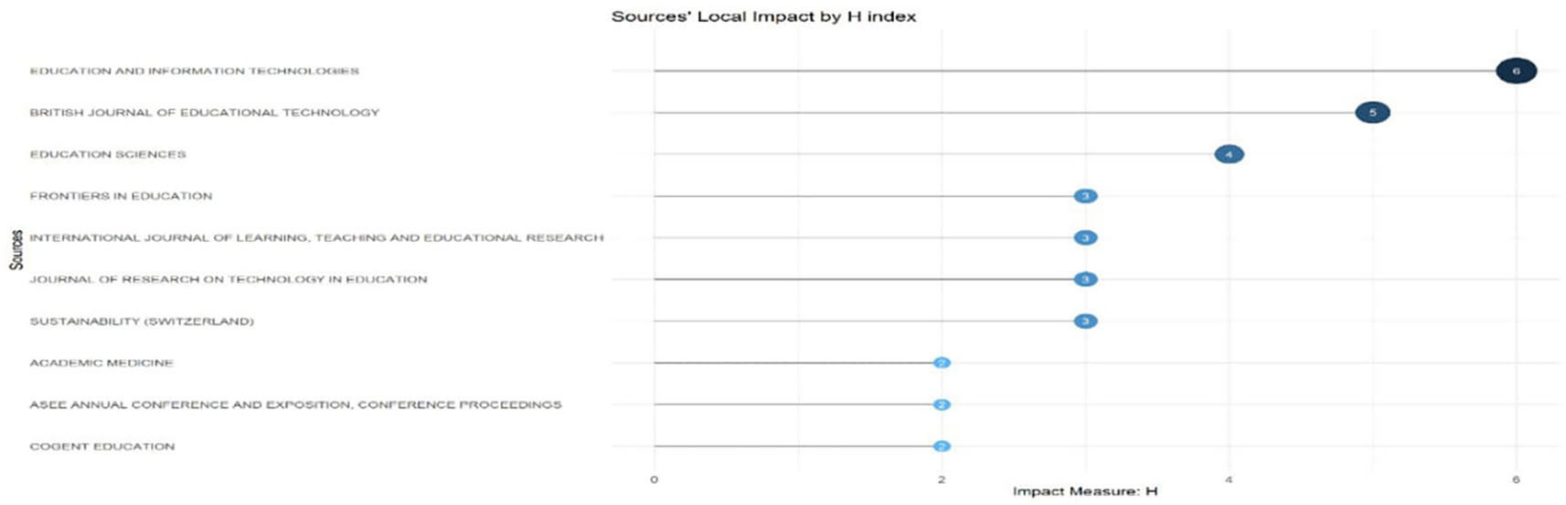
Sources’ local impact by H-index. Source: Processed data outcomes (2025).

**Figure 8.**
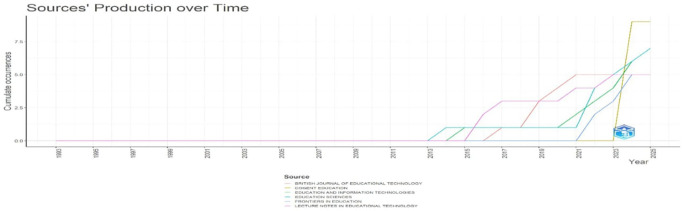
Sources’ production over time. Source: Processed data outcomes (2025).

Research question two investigates the primary sources publishing research on digital leadership in education from 2010 to 2024. Figure 5: Most Relevant Sources highlights the top journals contributing to the field, with Cogent Education leading in volume. Figure 6: Core Sources by Bradford’s Law identifies high-yield journals forming the publication nucleus. Figure 7: Sources’ Local Impact by H-index ranks these journals by citation influence, showing Education and Information Technologies as most impactful. Finally, Figure 8: Sources’ Production over Time illustrates cumulative output trends, revealing rapid growth in scholarly contributions, especially after 2015.

**Research question 2:** What are the sources of publications on digital leadership in education from 2010 to 2024?

Figure 5 shows a horizontal bar chart quantifying document contributions from key educational technology publication sources. Cogent Education leads with nine documents, followed by Education and Information Technologies and Education Sciences (7 each), British Journal of Educational Technology (5), Frontiers in Education (4), and Lecture Notes in Educational Technology (4) form the mid-tier contributors. The distribution demonstrates the interdisciplinary nature of educational technology research, with significant contributions from dedicated educational technology journals and broader education publications, revealing where the most substantial scholarly discourse occurs.

Figure 6, a graph, illustrates Bradford’s Law application to educational technology literature sources. The step-function plot maps the relationship between source logarithmic rank (x-axis) and article productivity (y-axis). The shaded “Core Sources” region identifies the most productive publications, with Cogent Education, Education and Information Technologies, and Education Sciences forming the highest-contributing nucleus. The distinctive downward trend illustrates the bibliometric principle that a small number of journals generate an excessively high proportion of pertinent articles. Researchers searching for high-impact publications in the field of educational technology can get empirical guidance from this visualisation, which successfully identifies the primary publication venues influencing this conversation.

Figure 7 represents the horizontal bar chart that maps the impact of local citations on educational technology publications through their H-index values. Education and Information Technologies leads with an H-index of 6, followed by British Journal of Educational Technology (5) and Education Sciences (4). Four sources share an H-index of 3 (Frontiers in Education, International Journal of Learning, Teaching and Educational Research, Journal of Research on Technology in Education, and Sustainability), while the three sources register an H-index of two. The visualisation shows the stratification of citation impact in this field, separating high-impact journals from those with more modest citation footprints in educational technology discourse.

Figure 8 shows the line graph, which tracks cumulative publication output from key educational technology journals between 1993 and 2025. The visualization reveals that significant publication activity only began around 2015, with dramatic acceleration after 2021. Cogent Education shows the steepest growth trajectory (yellow line), reaching approximately nine publications by 2025, while Education Sciences and Education and Information Technologies demonstrate substantial but more moderate growth. British Journal of Educational Technology and Frontiers in Education follow similar growth patterns. The temporal distribution indicates that educational technology has rapidly evolved as a research domain, with the most substantial scholarly contributions emerging within the past decade.

Research question three presents key findings about author productivity and impact in digital leadership research from 2010 to 2024, highlighting leading contributors and citation patterns that define the field’s scholarly landscape across key metrics such as: Most Relevant Authors, Authors’ Production over Time, Authors’ Local Impact by H-index, Most Globally Cited Documents and Most Locally Cited References.

**Research question 3**: What is the information on digital leadership about the authors with publications from 2010 to 2024?

**Figure 9.**
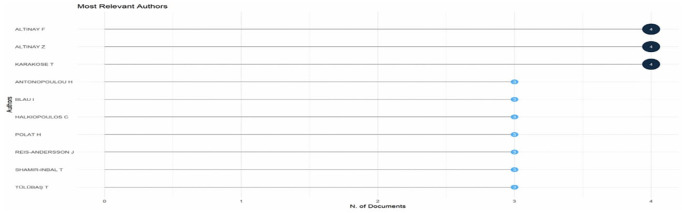
Most relevant authors. Source: Processed data outcomes (2025).

Figure 9 depicts the horizontal bar chart, quantifying publication productivity among key educational technology scholars. Three researchers lead with four publications each: Altinay F, Altinay Z, and Karakose T, establishing them as the most prolific contributors in this domain. Seven additional scholars (Antonopoulou H, Blau I, Halkiopoulos C, Polat H, Reis-Andersson J, Shamir-Inbal T, and Tulubas T) follow with three publications each. Similar publication counts suggest possible collaboration clusters, and the distribution shows a relatively small core of productive researchers driving educational technology scholarship. This productivity metric assists in identifying key players who influence conceptualisation and empirical research in the field.

**Figure 10.**
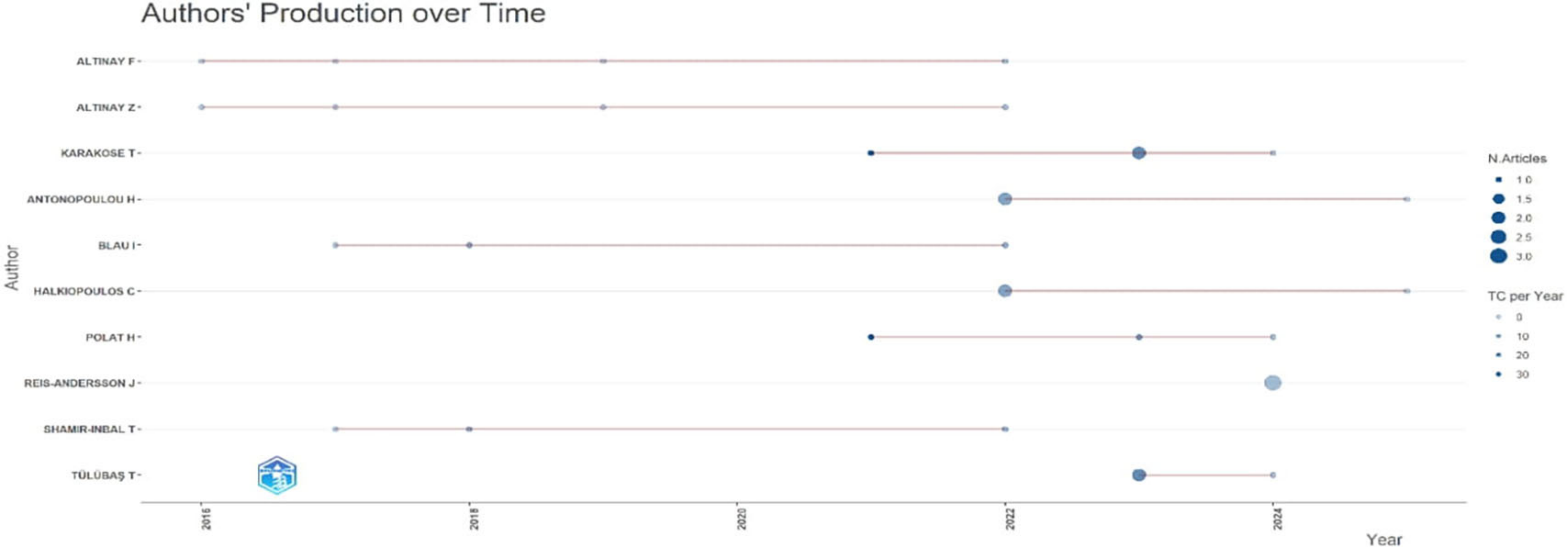
Authors’ production over time. Source: Processed data outcomes (2025).

The scatter plot from Figure 10 illustrates the temporal distribution and intensity of scholarly output among educational technology researchers from 2015 to 2024. Altinay F, Altinay Z, and Karakose T show the most sustained activity, with Altinay Z peaking in recent years. Marker size reflects the number of articles, while the vertical scale shows individual authors. Citation impact (TC per Year) varies, with Antonopoulou H and Tulubas T achieving higher influence per output in 2022 and 2023, respectively. The timeline reveals a growing research momentum post-2020, with visible clustering suggesting emerging collaboration patterns and thematic convergence.

**Figure 11.**
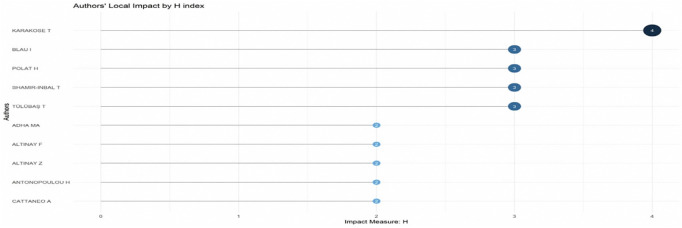
Authors’ local impact by H-index. Source: Processed data outcomes (2025).

The H-index scores of important educational technology researchers are displayed in a dot plot in Figure 11, emphasising their scholarly influence locally. Karakose T leads with an H-index of 4, indicating consistent citation of multiple publications. Four other scholars: Blau I, Polat H, Shamir-Inbal T, and Tulubas T share the next highest score of 3, reflecting solid citation performance across their output. A second tier of contributors, including Altinay F, Altinay Z, Antonopoulou H, and others, holds an H-index of 2 or below. The distribution underscores a small cohort of highly cited authors shaping localized academic discourse within the field.

**Figure 12.**
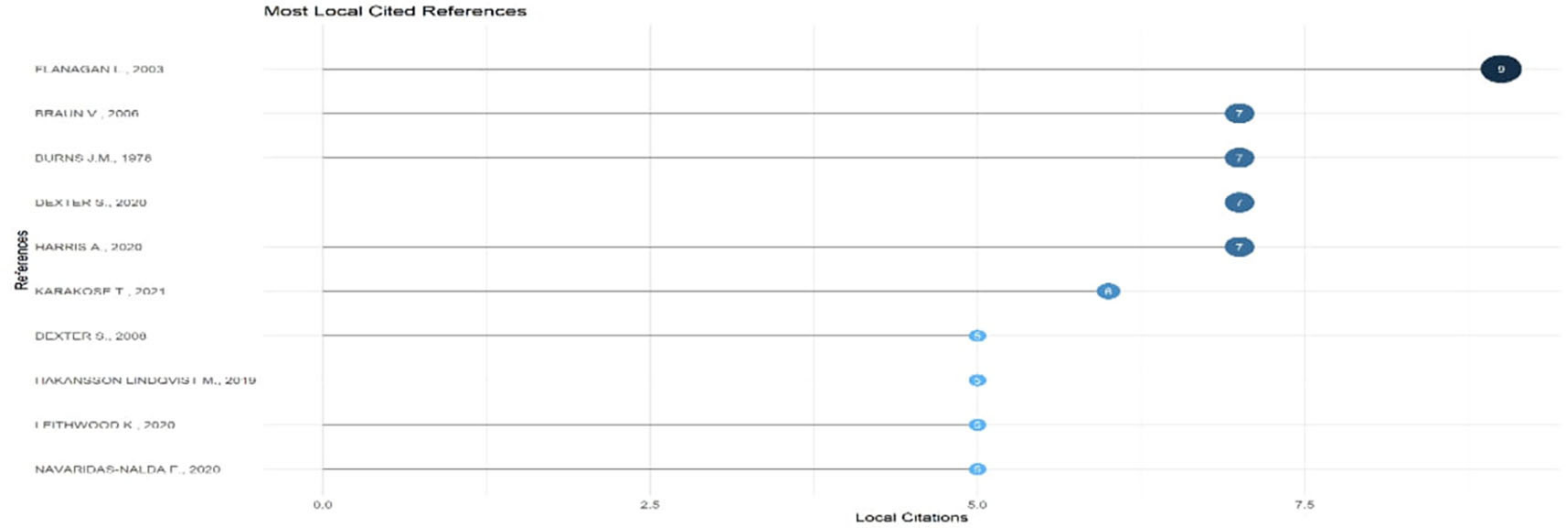
Most locally cited references. Source: Processed data outcomes (2025).

This horizontal bar chart in Figure 12 displays the most frequently cited references within the selected body of educational technology literature. Flanagan L. (2003) leads with nine local citations, followed closely by Braun V. (2006), Burns J.M. (1978), and Dexter S. (2020), each cited 7 times. These works likely represent foundational or widely applied theoretical frameworks. The presence of recent sources such as Karakose T. (2021) and Harris A. (2020) indicates the integration of contemporary discourse. The combination of older and modern citations reveals a scholarly dialogue that bridges long-established theories with emerging educational challenges.

**Figure 13.**
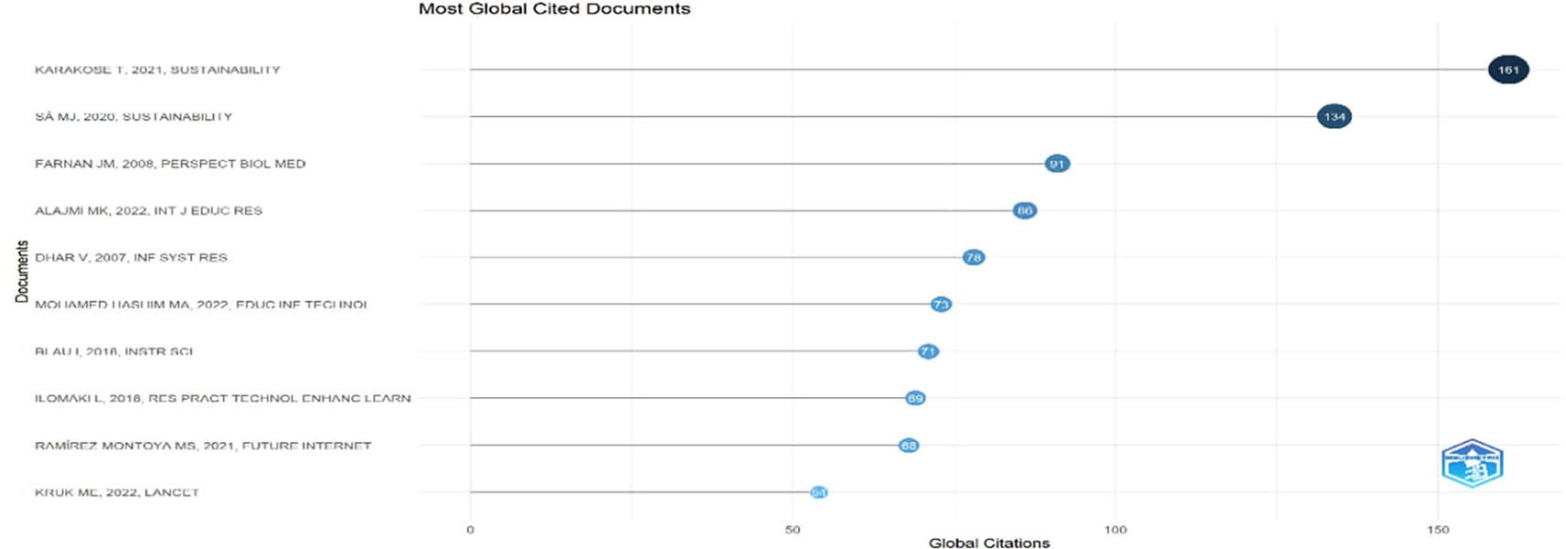
Most globally cited documents. Source: Processed data outcomes (2025).

This horizontal dot plot in Figure 13 ranks scholarly documents by total global citations within educational technology. Karakose T. (2021) tops the list with 161 citations, indicating substantial international influence, followed by Sá M.J. (2020) with 134. Farnan J.M. (2008) and Alajmi M.K. (2022) also show high visibility with 91 and 86 citations, respectively. The presence of both foundational medical education research and recent digital learning studies reflects interdisciplinary relevance. The citation patterns suggest growing global scholarly engagement with themes of sustainability, instructional innovation, and digital transformation in education.

**Figure 14.**
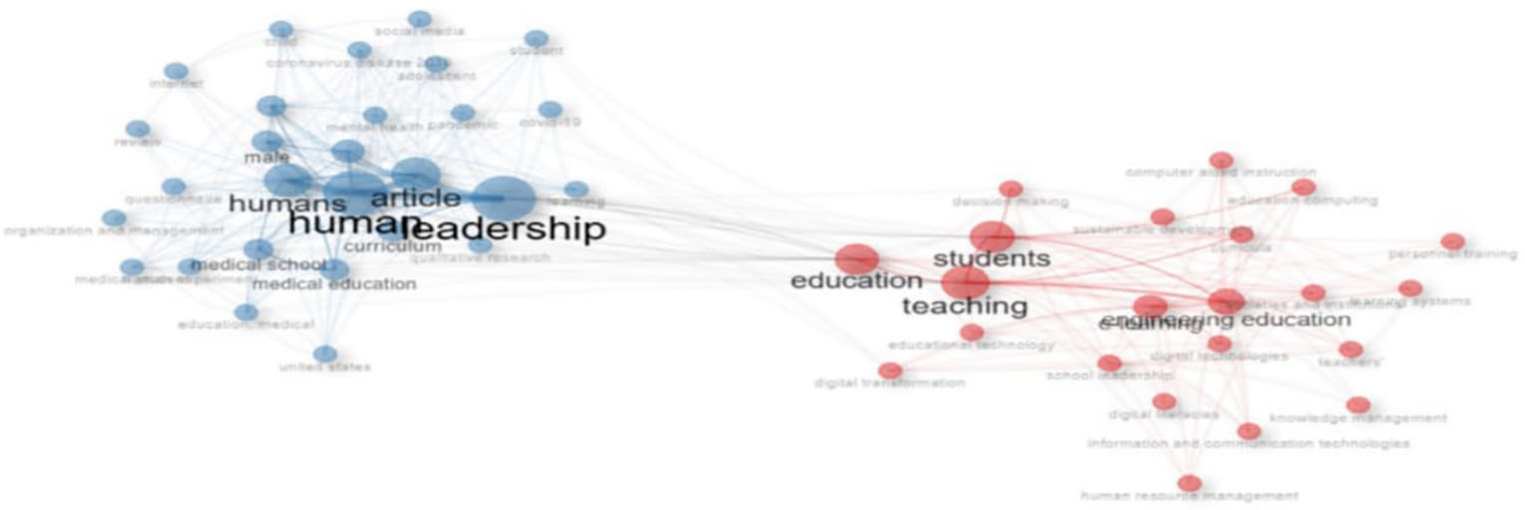
Co-occurrence network of keywords. Source: Processed data outcomes (2025).

**Figure 15.**
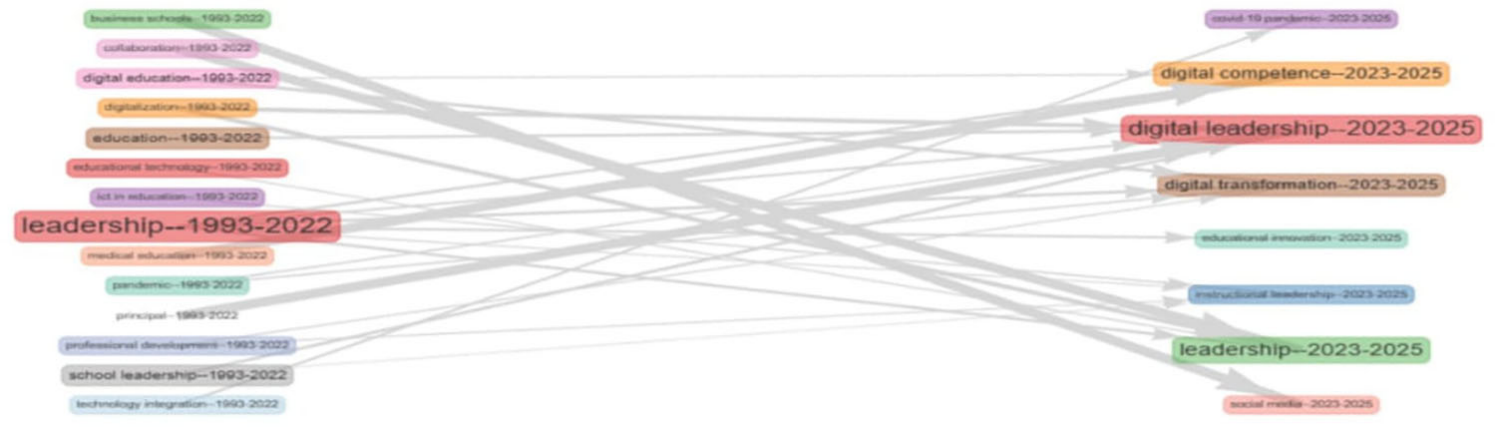
Thematic evolution of keywords. Source: Processed data outcomes (2025).

**Figure 16.**
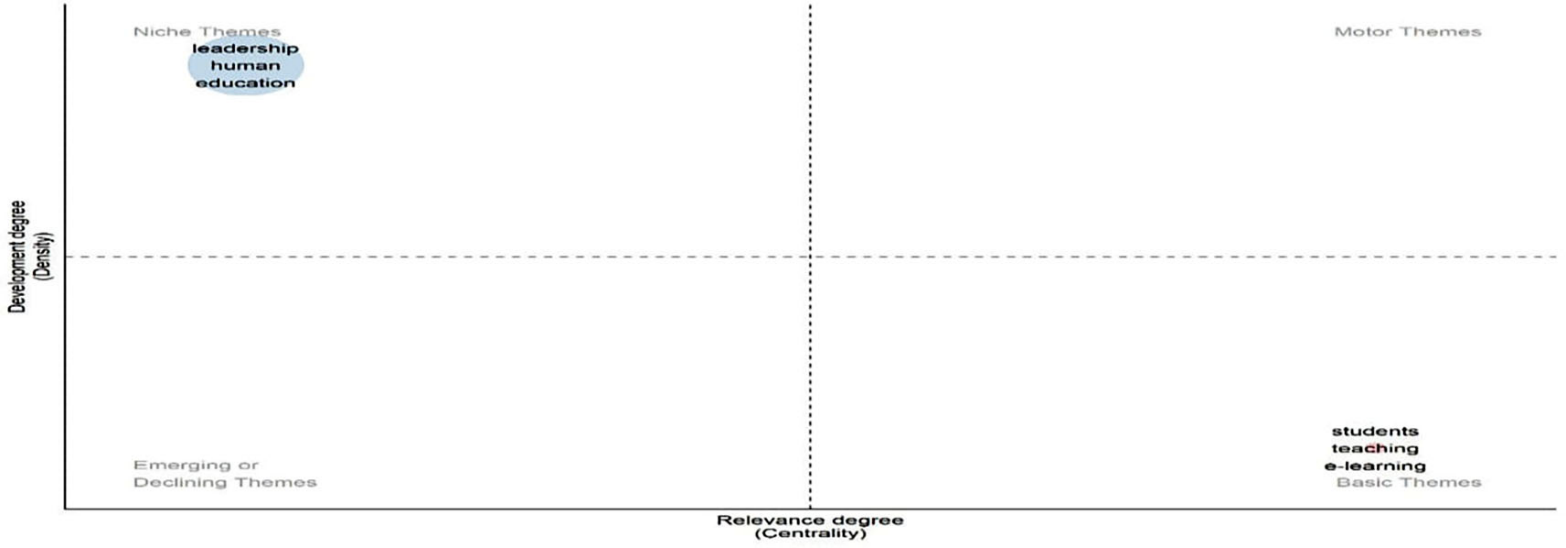
Thematic map of conceptual structure. Source: Processed data outcomes (2025).

**Figure 17.**
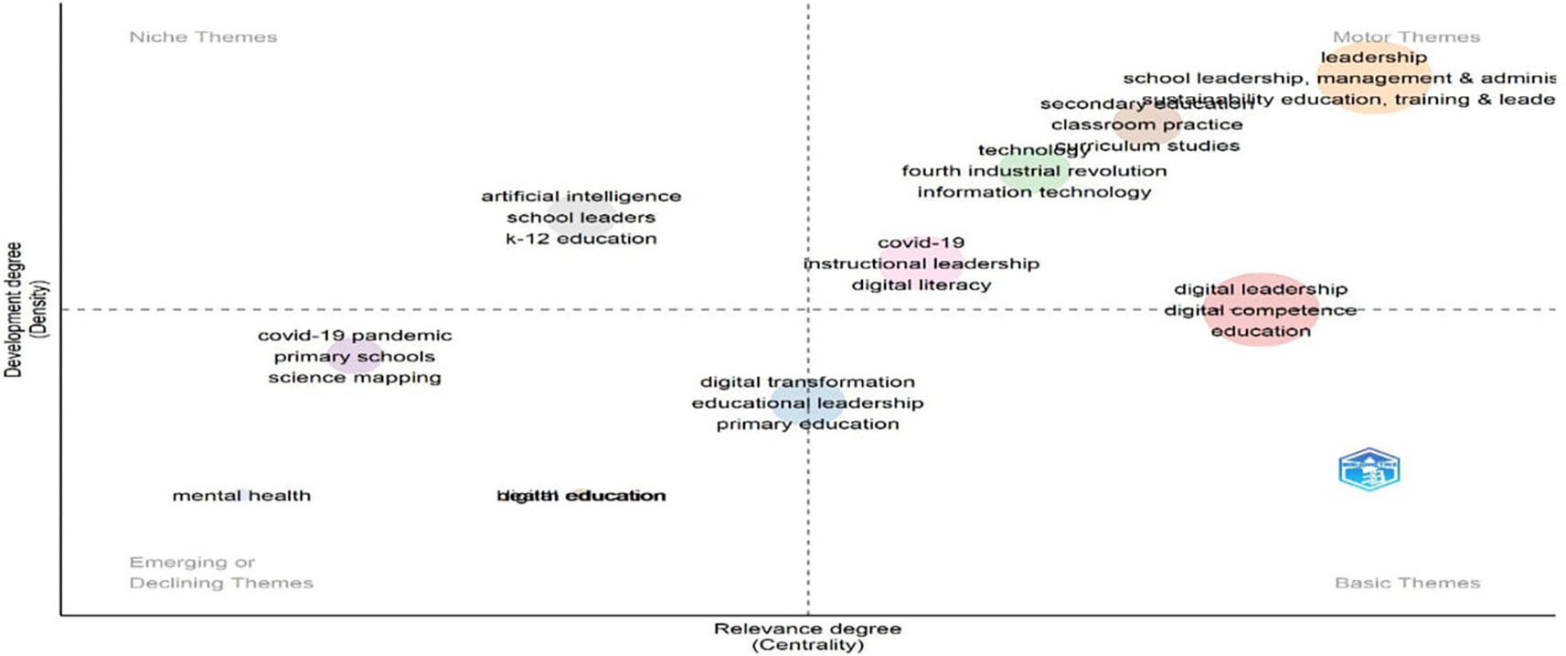
Strategic thematic map of educational leadership research. Source: Processed data outcomes (2025).

**Figure 18.**
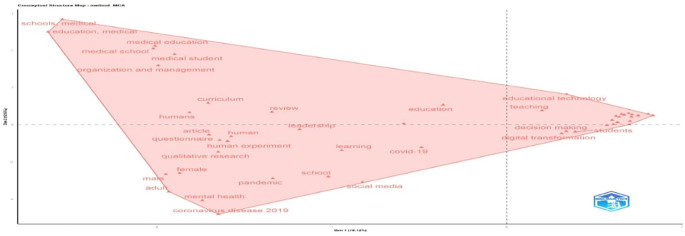
Conceptual structure map of educational research (MCA Method). Source: Processed data outcomes (2025).

**Figure 19.**
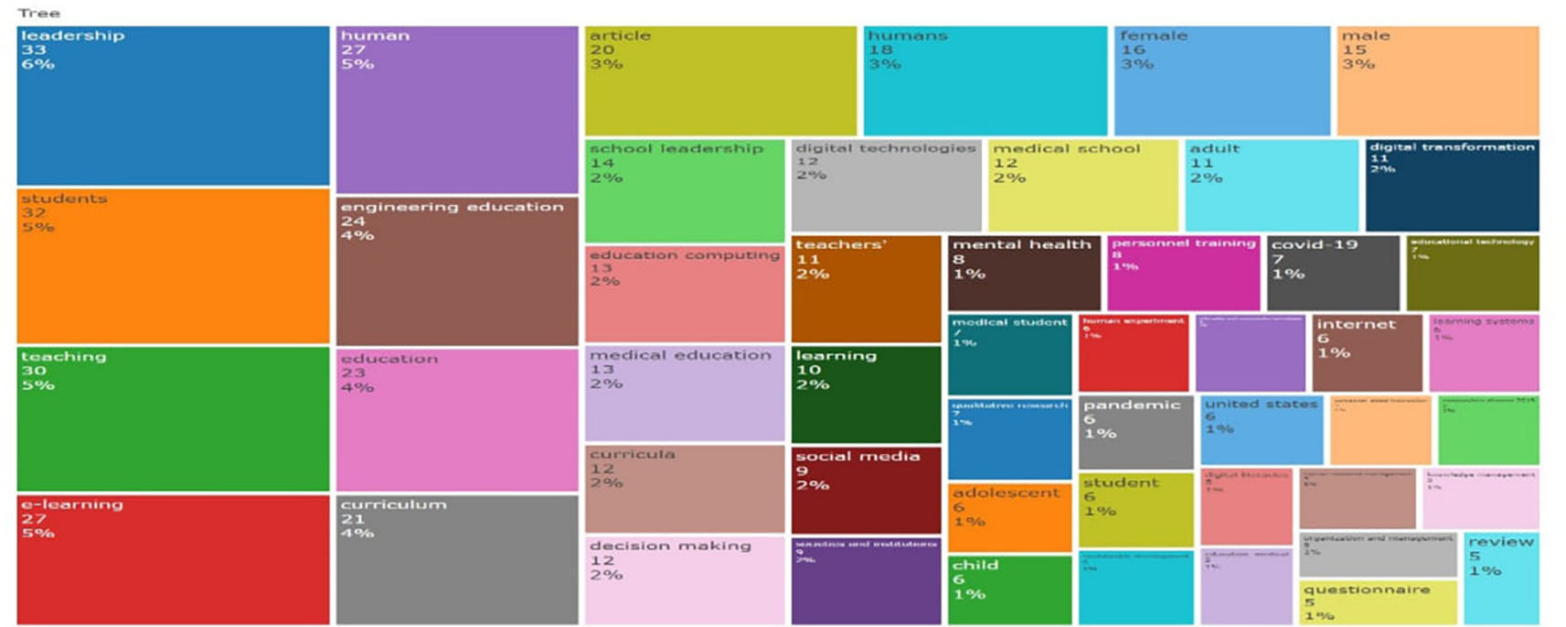
Keyword co-occurrence treemap. Source: Processed data outcomes (2025).

Research question four explores the conceptual structure of digital leadership in education from 2010 to 2024. Figures 14– 19 detail key themes, keyword dynamics, and conceptual trends of the study. From co-occurrence patterns ( Figure 14) to thematic evolution ( Figure 15) and strategic mapping ( Figures 16– 19), the findings reveal a field shaped by pedagogical, technological, and interdisciplinary concerns.

**Research question 4**: What are the most common themes, keywords, and concepts in digital leadership in education from 2010 to 2024?

This co-occurrence network visualizes thematic clusters within educational technology literature. Two major clusters emerge: the blue cluster emphasizes “leadership,” “humans,” and “medical education,” reflecting health sciences and organizational research; the red cluster centers on “students,” “teaching,” and “engineering education,” indicating pedagogical and digital learning themes. Bridging terms such as “education” and “teaching” suggests an interdisciplinary overlap. Node size denotes frequency, while edge thickness indicates keyword association strength. The network highlights the field’s bifocal structure—balancing leadership in health-related contexts and technological innovation in instructional environments.

Figure 15 illustrates the longitudinal shift in thematic focus within educational leadership research from 1993 to 2025. Early themes such as “leadership,” “school leadership,” and “digital education” (1993–2022) transition into contemporary emphases like “digital leadership,” “digital transformation,” and “educational innovation” (2023–2025). Notably, “leadership” remains central, evolving in scope and linking to future-focused constructs such as “digital competence” and “institutional leadership.” Thematic continuity is evident, yet the vocabulary increasingly reflects technological and pandemic-era concerns, signifying a conceptual pivot toward digitally mediated educational practices.

Figure 16 categorizes research themes by development (density) and relevance (centrality). “Leadership,” “human,” and “education” appear in the upper-left quadrant as well-developed niche themes, indicating specialization but limited influence across the broader field. On the other hand, “students”, “teaching”, and “e-learning” are grouped in the lower-right quadrant, indicating that they are fundamental themes that are underdeveloped despite being extremely central. These foundational topics play a key integrative role across studies but warrant further theoretical refinement. The absence of motor or emerging themes suggests a field stabilizing around established concepts, with limited current innovation or diversification.

Figure 17 identifies four thematic zones. “Leadership,” “school leadership,” and “management & administration” are motor themes—both central and well-developed, indicating strong influence and theoretical maturity. The concepts of “digital leadership”, “digital competence”, and “education” are fundamental yet essential; they need to be further developed to be as relevant as they are. Both “K-12 education” and “artificial intelligence” appear as niches, denoting focused but distinct research. In contrast, the lower-left quadrant contains the emerging or declining themes of “mental health” and “digital education,” indicating a lack of integration and growth. This distribution reflects a research field balancing traditional foundations with rising digital and contextual shifts.

The map in Figure 18 visualizes thematic clustering in educational research. The right cluster, dominated by “students,” “decision making,” and “educational technology,” highlights a strong digital and learner-centered focus. The central zone shows general terms like “education,” “learning,” and “leadership,” reflecting the field’s conceptual core. The left cluster is anchored in “medical education,” “schools,” and “organization,” indicating a distinct strand related to healthcare and institutional management. Terms on the lower left, like “mental health” and “coronavirus disease 2019”, show the influence of context. Overall, the structure shows a three-way research landscape that includes digital transformation, medical education, and pedagogy.

This treemap in Figure 19 visualizes the frequency and distribution of keywords across educational research literature. Dominant terms such as “leadership” (6%), “students” (5%), “teaching” (5%), and “e-learning” (5%) indicate sustained scholarly focus on pedagogy, digital environments, and organizational roles. “Engineering education”, “medical education”, and “curriculum”, which indicate sector-specific interests, are examples of mid-frequency terms. New trends are reflected in less common but noteworthy keywords like “digital transformation” and “social media.” The area-based, hierarchical layout emphasises thematic prominence and relational breadth, offering a thorough overview of research priorities and the changing field of educational inquiry.

**Figure 20.**
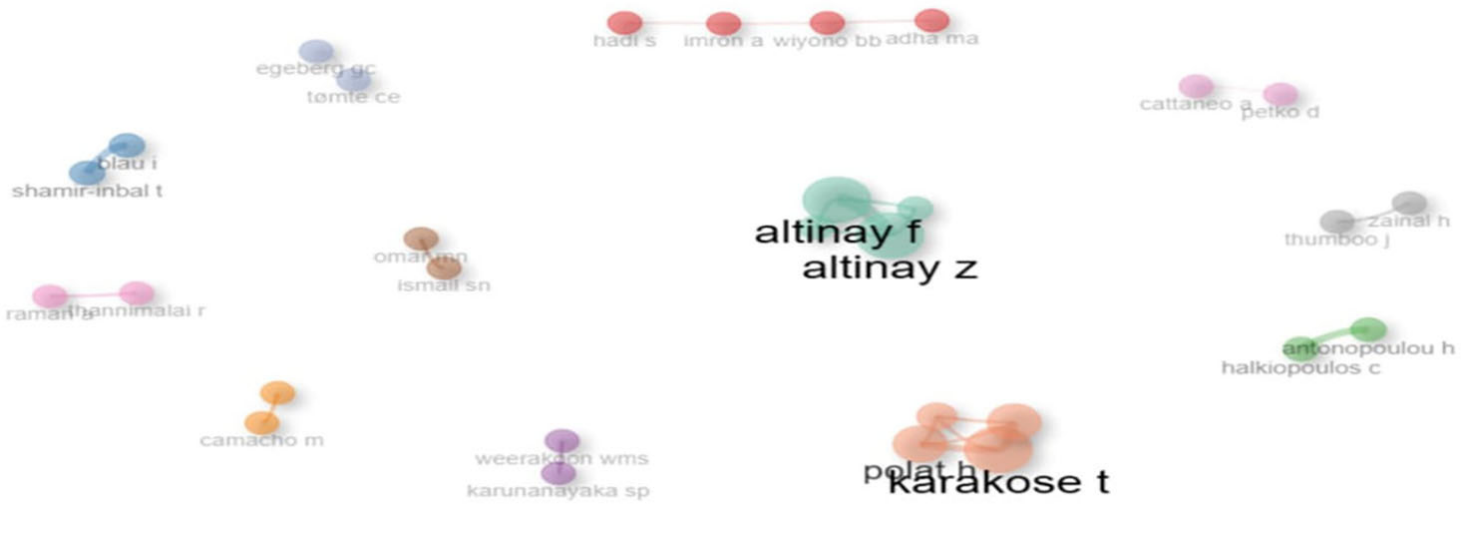
Co-authorship map. Source: Processed data outcomes (2025).

**Figure 21.**
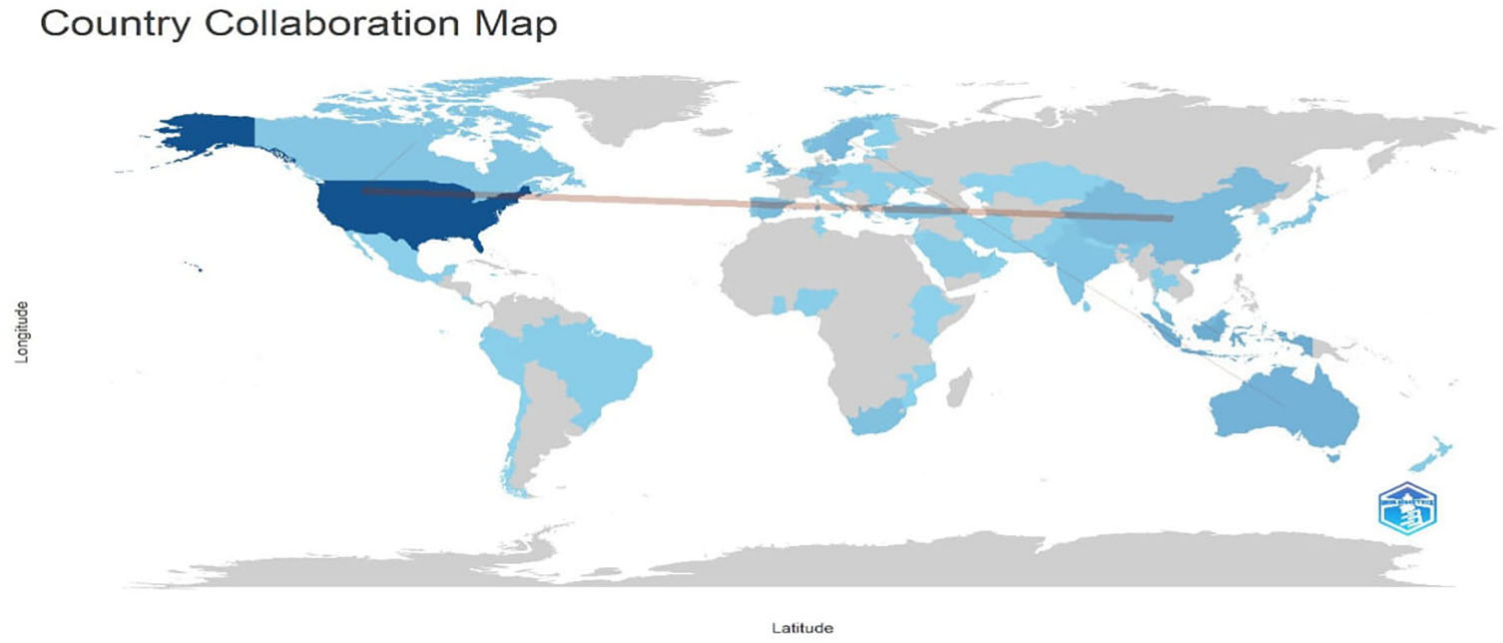
Country Collaboration Map (CCM). Source: Processed data outcomes (2025).

Research question five examines the social structure of digital leadership research in education between 2010 and 2024. Figures 20 and 21 visualize co-authorship and country collaboration patterns. The co-authorship map ( Figure 20) reveals key author networks, while the country collaboration map ( Figure 21) highlights global partnerships, led predominantly by developed nations.

**Research question 5**: What is the social structure of the publications on digital leadership in education from 2010 to 2024?

This co-authorship network in Figure 20 highlights collaborative clusters within educational research. Central figures such as Karakose T., Altinay F., and Altinay Z. display strong interconnections, indicating prolific joint contributions and central influence. Smaller, isolated clusters (e.g., Imron A. & Wiyono BB, Shamir-Inbal T. & Blau I.) suggest limited but focused collaborations. The size and proximity of nodes represent author prominence and partnership strength, respectively. The network underscores a fragmented yet collaborative scholarly landscape, with key authors forming distinct hubs of knowledge production.

The map in Figure 21 above illustrates patterns of international research collaboration, with the US standing out as the main hub and forming wide-ranging partnerships across continents. The United States has noteworthy partnerships with nations like Australia, the United Kingdom, and Turkey. Higher contributions are indicated by darker blue, which reflects varying degrees of research activity. The global distribution points to a concentration of collaborative networks in North America, Europe, and some parts of Asia-Pacific, underscoring regional differences and confirming the importance of developed countries in international academic collaboration.

## Discussion of findings

The bibliometric analysis of digital leadership in education from 2010 to 2024 revealed significant trends, thematic shifts, and collaborative patterns, addressing the five research questions guiding this study. Below is a structured discussion of the findings:

### Publication trends between 2010 and 2024

These findings show a significant increase in publications on digital leadership in education after 2010, with a peak in 2023 and a subsequent decline in 2024. The COVID-19 pandemic, AI breakthroughs, and the need for digital leadership skills have all contributed to the global acceleration of digital transformation, especially in education. The recent steep decline may indicate saturation or a change in research priorities, whereas the surge reflects increased scholarly interest in how digital tools transform educational leadership. The citation trends reflected the growth in publications, highlighting the field’s growing impact. Recent studies corroborate this study’s findings that there was a significant surge in publications post-2010 on digital leadership research in education ( Adeoye et al., 2023; Wollscheid et al., 2025; Zhao & Zhou, 2024).

### Publication sources between 2010 and 2024

A concentration of influential publications in a few core journals, such as Cogent Education, Education and Information Technologies, and Education Sciences, emerged as central outlets for digital leadership research. Bradford’s Law confirmed these as core sources, with Education and Information Technologies exhibiting the highest local impact (H-index = 6). The dominance of these journals highlights their role in disseminating cutting-edge research, while the rapid post-2015 growth in output suggests the field’s maturation as a distinct research domain. Some scholars attest to an increasing concentration of publications in key journals and the development of thematic areas over the reference time ( Adeoye et al., 2023; Karakose et al., 2024; Makda, 2025; Wollscheid et al., 2025).

### Author productivity and impact between 2010 and 2024

The results highlight a compact group of prolific scholars consistently demonstrating volume and influence in digital leadership education discourse. Karakose T., Altinay F., and Altinay Z. emerge as high-impact contributors, with Karakose demonstrating the highest local influence (H-index = 4) and notable global citations (e.g., Karakose et al., 2021 study with 161 citations). According to previous bibliometric studies, Karakose is an important contributor to the discourse on digital leadership, primarily as a result of collaborative efforts to analyse the field’s theoretical limitations and evolution ( Karakose et al., 2022; Karakose & Tülübaş, 2023). Similar to this, Altinay F. and Altinay Z.’s joint scholarship shows a strong emphasis on inclusive digital leadership practices, especially in the areas of educational accessibility and sustainability ( Baglama et al., 2022). The analysis also demonstrates the field’s increasing intellectual cohesion and thematic maturity. Notably, post-2020 co-authorship networks show intensified collaboration, likely driven by global digital disruptions such as COVID-19, accelerating the transition to remote research and leadership models. The interplay between foundational leadership frameworks (e.g., Burns, 1978) and emergent digital competencies ( Dexter et al., 2020; Harris & Jones, 2020) highlights how established theories are adapted to meet contemporary educational challenges.

### Conceptual structure between 2010 and 2024

The conceptual structure, as revealed through co-occurrence analysis and thematic mapping, points to a dual orientation toward educational leadership principles and digital transformation technologies. Through a bibliometric mapping approach, Karakose et al. (2022) demonstrate that digital competence, virtual leadership, and innovation have become core thematic clusters, particularly after 2020, coinciding with pandemic-induced shifts in educational delivery. Thematic evolution and strategic mapping further underscore a maturing research landscape, with shifts from technology management function to a more holistic construct encompassing AI integration and institutional digital capacity ( Sagbasi & Erdogan, 2022). Similarly, Magesa and Jonathan (2022) and Gilli et al. (2023) confirm that thematic evolution showed a post-pandemic pivot toward digital transformation leadership in response to systemic challenges, underlining the global relevance of digital competencies across institutional contexts. The lack of motor themes, however, suggests that the field is maturing around central concepts, with little innovation in specialised fields like artificial intelligence and K–12 education.

### Social structure between 2010 and 2024

The social structure of contemporary educational digital leadership research between 2010 and 2024 reveals a fragmented yet expanding scholarly collaboration network. Co-authorship patterns indicate the emergence of closely connected author clusters, particularly among prolific contributors such as Karakose and Altinay, whose frequent collaborations exemplify regional academic cohesion ( Wollscheid et al., 2025). Globally, countries like the United States, Turkey, and the United Kingdom dominate as research hubs, reflecting their roles and influences in knowledge production in the Global North ( Tigre et al., 2023). This study also aligns with Chasokela et al. (2025), who contended that digital technologies have facilitated international research exchange but reinforce the structural disparities in global participation. Consequently, developing nations continue to be under-represented in high-impact publishing networks, indicating that initiatives aimed at enhancing capacity and promoting digital equity are essential for inclusive knowledge production in the field of digital leadership.

## Conclusion

A thorough review of the research trends in digital leadership in education from 2010 to 2024 is given by this bibliometric analysis, which also shows a notable increase in scholarly output, thematic evolution, and collaborative networks. The study draws attention to a dramatic rise in publications after 2010, which peaked in 2023 and demonstrated how digital leadership is increasingly acknowledged as a crucial and developing field of study in educational research. The analysis also reveals a concentration of research within selected high-impact journals such as Education and Information Technologies and Cogent Education, while the dominance of scholars like Karakose and Altinay indicates the emergence of thought leaders shaping the discourse. Especially in post-pandemic educational settings, the research’s conceptual structure, as shown by thematic mapping, showcases how the field is moving from generic digitalisation narratives to complex constructions, including artificial intelligence integration, digital competencies, virtual leadership, and institutional innovation. Nevertheless, the drop in publications in 2024 points to possible saturation or changing research priorities, prompting more investigation. The analysis of the social structure reveals a dispersed but developing network of cooperation. The study shows continuing variations in contribution between the Global North and South in spite of rising inquiry volume and global collaboration. Though countries like the United States, the UK, and Turkey show great impact, much of the Global South remains under-represented. This splintered social framework emphasises the need for expansive, inclusive collaborative research and a deliberate concentration on digital equity to ensure that educational leadership development reflects global realities rather than just the interests of leading economies. The outcomes also underline how global events like the COVID-19 epidemic hasten digital transformation research and support fresh leadership models. In summary, this study delineates the intellectual terrain of digital leadership in education, laying the foundation for future research and practical applications.

### Implications of the study

The study’s conclusions have profound implications for academics, policymakers, and educational leaders. The trends that have been identified show scholars that more interdisciplinary research is needed in the field of digital leadership, with new technologies like artificial intelligence, machine learning, and data analytics in education. Thematic gaps offer opportunities for further research to advance novel concepts. Additionally, the dominance of research centres in the Global North demands greater equity in knowledge creation, thereby promoting collaboration that clearly stands up for underrepresented regions. In conclusion, expanding global research networks may contribute to a more equitable and diverse discourse of leadership in the digital era in education.

For policymakers, the study emphasises the need for them to include digital leadership competencies in professional development plans for teachers and school administrators. Policies should accord capacity-building projects top priority given the rapid digital transformation in education since they will equip leaders with the tools to handle technological interruptions. Moreover, differences in global research participation point to the need for financing and infrastructure support to improve digital scholarship in developing countries. Policymakers can guarantee that educational institutions globally are better ready for future obstacles by supporting inclusive digital leadership ecosystems.

For educational leaders, the results highlight how important it is to use adaptive leadership techniques that keep up with technological developments. To effectively use emerging technologies and guarantee that digital transformation leads to improved teaching and learning outcomes, educational leaders must embrace continuous learning. The study highlights the value of cooperative networks and exhorts educational leaders to engage in knowledge-sharing forums that promote digital leadership best practices. Stakeholders can promote substantial innovation in education and prepare institutions for a future that is increasingly focused on technology by resolving the shortcomings and applying the knowledge gained from this study.

## Ethical consideration

All the included articles are publicly accessible online and have been appropriately cited, and as a result, this study did not require ethical consideration.

## Data Availability

The datasets generated for Digital Leadership in Education: A Bibliometric Analysis of Research Trends from 2010 to 2024 are openly available in https://doi.org/10.5281/zenodo.15661591 ( Okunlola and Naicker, 2025). This project contains the following underlying data:
1. Digital Leadership in Education: A Bibliometric Analysis of Research Trends from 2010 to 2024 Scopus Data. Data are available under the terms of the Creative Commons Attribution 4.0 International license (CC-BY 4.0).
