# Supplementary material for: Digital Leadership in Education: A Bibliometric Analysis of Research Trends from 1993 to 2024
Source: F1000Res. 2025 Sep 19;14:687. Originally published 2025 Jul 11. [Version 2] doi: 10.12688/f1000research.166667.2 (PMC12475901; doi:10.12688/f1000research.166667.2)
Supplement: Peer review history

# Reviewer report: Reviewer response for version 2

**DOI:** 10.5256/f1000research.188151.r416211

**Published:** 2025-09-27

**Authors:** Sulaimon Adewale

**Affiliations:** 1 Department of Educational Foundation, University of South Africa, Pretoria, South Africa

The study is deemed technically sound, employing an appropriate bibliometric methodology. The underlying data is openly available, ensuring reproducibility. However, the review identifies key areas for improvement. The work is only partly clearly presented due to an over-reliance on citations from a narrow group of authors and occasional complex sentence structures. While the methodology is appropriate, conclusions are only partly supported, as some policy recommendations extend beyond the direct bibliometric evidence.

Is the work clearly and accurately presented and does it cite the current literature?

Yes

If applicable, is the statistical analysis and its interpretation appropriate?

Yes

Are all the source data underlying the results available to ensure full reproducibility?

Yes

Is the study design appropriate and is the work technically sound?

Yes

Are the conclusions drawn adequately supported by the results?

Partly

Are sufficient details of methods and analysis provided to allow replication by others?

Partly

Reviewer Expertise:

Education leadership and management, peace studies and gender studies.

I confirm that I have read this submission and believe that I have an appropriate level of expertise to confirm that it is of an acceptable scientific standard.

# Reviewer report: Reviewer response for version 2

**DOI:** 10.5256/f1000research.188151.r415532

**Published:** 2025-09-26

**Authors:** Moses Adeleke ADEOYE

**Affiliations:** 1 Al-Hikmah University, Ilorin, Nigeria

I have carefully considered the revised manuscript and the authors’ detailed responses to earlier peer review reports. The revisions have addressed many of the concerns previously raised, particularly through:

- Clarification of methodological details, including software packages, Bibliometrix/biblioshiny interface, and threshold parameters.
- Correction of date inconsistencies and improvement of abstract accuracy.
- Integration of COVID-19’s impact and references that strengthen contextual framing.
- Expanded implications for practice and policy, making the work more actionable.

The bibliometric scope remains focused on education, which the authors have justified as a deliberate and bounded choice. While broader comparisons (e.g., with public sector or government-led digital transformation) could have added further insights, I accept the authors’ clarification that such extensions fall beyond the present study’s intent and may be pursued in future work.

 Overall, the manuscript is now more rigorous, coherent, and transparent. The study makes a useful contribution by mapping the intellectual and social structure of digital leadership in education, while also highlighting the persistent gap in Global South participation.

**Final Position:** I have no further substantive comments to make. I consider the manuscript suitable for indexing in its current form.

Is the work clearly and accurately presented and does it cite the current literature?

Partly

If applicable, is the statistical analysis and its interpretation appropriate?

Partly

Are all the source data underlying the results available to ensure full reproducibility?

Yes

Is the study design appropriate and is the work technically sound?

Partly

Are the conclusions drawn adequately supported by the results?

Partly

Are sufficient details of methods and analysis provided to allow replication by others?

No

Reviewer Expertise:

Educational Leadership

I confirm that I have read this submission and believe that I have an appropriate level of expertise to confirm that it is of an acceptable scientific standard.

# Reviewer report: Reviewer response for version 2

**DOI:** 10.5256/f1000research.188151.r415533

**Published:** 2025-09-26

**Authors:** Asem Obied

**Affiliations:** 1 Palestine Technical University-Kadoorie, Ramallah, Palestinian Territory

good job

Is the work clearly and accurately presented and does it cite the current literature?

Yes

If applicable, is the statistical analysis and its interpretation appropriate?

Yes

Are all the source data underlying the results available to ensure full reproducibility?

Yes

Is the study design appropriate and is the work technically sound?

Yes

Are the conclusions drawn adequately supported by the results?

Yes

Are sufficient details of methods and analysis provided to allow replication by others?

Yes

Reviewer Expertise:

NA

I confirm that I have read this submission and believe that I have an appropriate level of expertise to confirm that it is of an acceptable scientific standard.

## References

[1] : The professional competence of faculty members from the students’ perspective at Kuwait University and Palestine Technical University Kadoorie. *Arab Gulf Journal of Scientific Research* .2024;42(4) : 10.1108/AGJSR-05-2023-0208 1755-1769 10.1108/AGJSR-05-2023-0208

# Reviewer report: Reviewer response for version 1

**DOI:** 10.5256/f1000research.183690.r404289

**Published:** 2025-08-26

**Authors:** Elizabeth Valentine

**Affiliations:** 1 Victoria University of Wellington, Wellington, Wellington, New Zealand

*Peer Review of Digital Leadership in Education: A Bibliometric Analysis of Research Trends from 2010 to 2024*

 Summary:

 This article applies biometric analysis to answer five questions pertaining to trends in Digital Leadership (DL) in education:

- 1. What is the trend in the number of publications on digital leadership in education from 2010 to 2024?
- 2. What are the sources of publications on digital leadership in education from 2010 to 2024?
- 3. What is the information about the authors with publications from 2010 to 2024?
- 4. What are the most common themes, keywords, and concepts on digital leadership in education from 2010 to 2024?
- 5. What is the social structure of the publications on digital leadership in education from 2010 to 2024?

The emphasis is on publication numbers, sources, authors and the social structures with a small amount of analysis on themes and trends based on the other question foci.

 Comments

 While on face value this article appears academically sound, I found the work lacking rigour for three reasons:

 First, DL has not ‘newly emerged’ neither is it a ‘new necessity’ (para 1).

 The term "digital leadership" was first coined in 2002 by Fisk to describe a new form of leadership style essential for navigating digital transformation. Further, the related concept of "e-leadership" was introduced earlier by Avolio in 2000, signifying leadership mediated by digital technologies. Before that, in education we had decades of scholarly research grounded in e-Learning.

 Further, more recently, DL has been more widely described as a strategy and an outcome (e.g., Adie et al, Westerman et al). These additional but important distinctions have also not been made in this work. Both are applicable to education.

 What would have added real value are trend-based insights for those responsible for  making DL and Digital Transformation (DT) decisions when the requirement to transform at scale and at pace (Adie et al) is faced by most (all?) education institutions, at all levels.

 Second, to add anything substantial to the body of knowledge, the article seems too narrowly focused on education i.e., it fell into the trap of being solely in an education silo. Thus, I suggest there are missed opportunities and reduced insights by not marginally expanding the context within which the education sector is strategized, funded and monitored. As a consequence, I suggest the search questions and application of the excellent methodology were also narrow. For example, the search might have added then compared and contrasted digital transformation in government. (Education is most commonly a part of the public wider sector).

 Third, this work does not clarify the two major, separate but interdependent areas of DT in education.

 While hinted at in terms of ‘operations’ and learning and teaching delivery the work lacks clarity between these two aspects, which in turn reduces the ability to more clearly articulate a DL *capability* focus (and to better identify and target competencies).

 It could have added real value to the research questions had the emphasis been to identify and made clear distinctions between running an education institution as a *business* (from governance, strategic, financial and education performance and compliance perspectives) and research highlighting excellence in teaching and learning as a result of DT.

 For some time and in practice, the interplay between school operations and administration, and the design, delivery, monitoring and measurement of education results has become fundamental to successful DL and DT approaches, investment, and prioritization. With clear distinction it is then possible to articulate strategy-matching capabilities and associated competencies (as the building blocks of capability (Valentine 2016 - 2026).

 Suggested improvements **:**

1. Covid 19 is mentioned but the impact and consequences for the transformation of technology use in education is neither summarized nor explored.  A quick Google Scholar search provided the following:
  [reference1], [reference 2], [reference 3]
  My comments also apply to references to the 4 ^th^ Industrial Revolution and the current and emerging use of AI in education. 
2. Therefore, while the research questions appear sound, greater and therefore deeper, more actionable insights might have been gained had questions been focused on understanding the impacts of 4LR, COVID and AI, and what those education institutions that were identified as ‘Digital Leaders’ as an outcome, and therefore held up as benchmarks; and questions about or those who provided Digital Leadership (as a role with accountabilities) and what each did differently in response to 4LR, C19 and AI. This would have enabled insights about the strategy-matching capabilities and competencies each developed to transform.
3. A review of literature associated with the education value chain which prioritizes the student journey and quality pedagogy while also underpinning these with the business, data and technology architecture required, is where things have been heading for some time, from DT and therefore DL perspectives.
  This dual focus is required to deliver strategies, business / operating and academic plans, financial and education performance and compliance outcomes.

Is the work clearly and accurately presented and does it cite the current literature?

Partly

If applicable, is the statistical analysis and its interpretation appropriate?

Not applicable

Are all the source data underlying the results available to ensure full reproducibility?

No source data required

Is the study design appropriate and is the work technically sound?

Partly

Are the conclusions drawn adequately supported by the results?

Partly

Are sufficient details of methods and analysis provided to allow replication by others?

Yes

Reviewer Expertise:

ICT in Education, IT Governance Competencies for Boards and Senior Leaders; Digital Leadership; Digital Transformation; Capability and Competency in Leadership.

I confirm that I have read this submission and believe that I have an appropriate level of expertise to state that I do not consider it to be of an acceptable scientific standard, for reasons outlined above.

## References

[1] : How the COVID-19 Pandemic Is Reshaping the Education Service. 10.1007/978-981-33-4126-5_2 15-36 10.1007/978-981-33-4126-5_2

[2] : Digital Transformation and Technological Innovation on Higher Education Post-COVID-19. *Sustainability* .2023;15(3) : 10.3390/su15032466 10.3390/su15032466

[3] : COVID-19 and the Digital Transformation of Education: What Are We Learning on 4IR in South Africa?. *Education Sciences* .2020;10(7) : 10.3390/educsci10070180 10.3390/educsci10070180

# Response

**Published:** 2025-09-13

**Authors:** John Olayemi OKunlola

**Affiliations:** educational leadership and mgt, University of Johannesburg, Auckland Park, Gauteng, South Africa

- The study applies **a bibliometric approach as its guiding framework** **, **offering both methodological and conceptual grounding. Since bibliometric analysis itself functions as a theoretical lens, additional frameworks may not be strictly necessary.
- Thank you for this valuable feedback. We acknowledge the comment and have expanded the *implications of the study* section to provide actionable recommendations for policymakers and practitioners.
- The inaccuracy in the usage of the years 1993 and 2010 has been corrected.
- Identified gaps in the abstracts have been re-examined and catered for.
- “The methodological section outlines the use of RStudio and bibliometric analysis adequately, but fails to provide specific information on the software packages or analysis thresholds”. This has been treated in the methodological section as identified and suggested.
- Figure 4: The Sankey Citation Flow Map was appropriately integrated into the main manuscript. Perhaps a mismatch occurred during copy-editing.
- In this study, **maturity denotes thematic coherence and growing intellectual cohesion, not conceptual closure** **.** The findings show sustained evolution, as emerging themes like AI integration and institutional digital capacity continue to expand the field’s boundaries.
- We appreciate this constructive observation. While the manuscript drew significantly on prolific authors such as Karakose and Altinay, this was due to their high visibility in the bibliometric dataset. As a bibliometric-driven study, the analysis is inherently bounded by the available data indexed in Scopus, which naturally foregrounds the most productive and frequently cited authors.
- We thank the reviewer for this observation. The manuscript did state the use of RStudio and *bibliometrix* *,* though not explicitly. For clarity, we now specify that the analysis was conducted using the *biblioshiny* interface of *bibliometrix*, with thresholds and clustering parameters applied accordingly.
- The manuscript has been revised to include the search strings, field tags, Boolean operators, and parameter thresholds used in RStudio, ensuring methodological transparency and reproducibility.
- In this study, **maturity denotes thematic coherence and growing intellectual cohesion, not conceptual closure** **.** The findings show sustained evolution, as emerging themes like AI integration and institutional digital capacity continue to expand the field’s boundaries.
- We sincerely appreciate the reviewer’s thoughtful suggestion. However, the focus of this study is deliberately confined to **digital leadership in education**, as this represents both the authors’ research niche and the central objective of the paper. While expanding to broader public-sector transformations (e.g., government or health) could generate comparative insights, it would risk diluting the study’s primary purpose: to map, analyze, and advance understanding of digital leadership specifically within the educational sector. We therefore retain this focus to ensure alignment with the study’s aims, while noting that future research may extend the methodology to cross-sectoral contexts.
- We appreciate this insightful comment. Indeed, the **education value chain** linking pedagogy, student experience, and institutional processes with data and technology architectures is an important dimension of digital transformation. However, the present study’s scope is confined to digital leadership within the school system. To avoid diluting its focus, we did not explicitly extend into business or financial architectures. Nonetheless, we agree that future research could productively integrate this dual focus by exploring how digital leadership mediates the intersection of pedagogy with institutional strategy, performance, and compliance outcomes.
- As suggested, the impact of COVID-19 on digital transformation in education has now been incorporated and supported with references to Kang (2021), Deroncele-Acosta et al. (2023), and Mhlanga & Moloi (2020)

# Reviewer report: Reviewer response for version 1

**DOI:** 10.5256/f1000research.183690.r401740

**Published:** 2025-08-25

**Authors:** Moses Adeleke ADEOYE

**Affiliations:** 1 Al-Hikmah University, Ilorin, Nigeria

1. Summary of the Article

 The manuscript titled “Digital Leadership in Education: A Bibliometric Analysis of Research Trends from 2010 to 2024” by John Olayemi Okunlola and Suraiya Rathankoomar Naicker presents a bibliometric analysis of 338 Scopus-indexed publications using RStudio. It evaluates publication trends, author productivity, conceptual themes, and collaborative structures related to digital leadership in educational contexts. The study identifies key publication sources, highlights dominant research themes like digital competence and AI integration, and uncovers global collaborative patterns—emphasising gaps in Global South participation. The paper aims to establish a foundational intellectual map to guide further research, policy, and practice.

 2. Evaluation of Scientific Soundness

 Title and Abstract

 The title accurately reflects the study's content. The abstract provides a broad overview, but terms like 'fragmented collaborations' or 'institutional innovation' need clarification. The abstract could be more precise in stating the context behind peaks or declines in publication trends.

 Introduction

 The introduction presents relevant background information but tends to repeat citations of particular authors (e.g., Karakose) without critical differentiation. Several sentences are grammatically awkward and could benefit from copy-editing. Clearer articulation of the research gap is needed.

 Literature Review

 While the review includes current and relevant literature, it lacks subheadings and a synthesized thematic structure. The discussion often reads like an introduction. An improved structure and clearer articulation of theoretical gaps would enhance its value.

 Methodology

 The methodological section outlines the use of RStudio and bibliometric analysis adequately, but fails to provide specific information on the software packages or analysis thresholds (e.g., clustering methods, minimum citation counts). More transparency is needed for reproducibility.

 Results

 The analysis is rich in visuals and quantitative detail, but some figures (e.g., Sankey maps) are not well-integrated into the discussion. Result narratives often drift into interpretive commentary. The presentation should distinguish more clearly between results and discussion.

 Discussion

 The discussion demonstrates a good grasp of scholarly trends and contextual relevance but could diversify its source base. Over-reliance on a few scholars skews the analysis. Deeper comparative engagement with existing studies would improve depth.

 Conclusion and Implications

 A strong summative section, but the term 'mature field' is used without sufficient evidence. Clarify whether maturity refers to volume, thematic coherence, or conceptual closure. Reframe to highlight ongoing evolution and emerging areas.

 Language and Style

 Generally readable but marked by long, complex sentences and minor grammatical errors. Copy-editing is needed for clarity, tone, and conciseness.

 Ethics and Data

 All ethical considerations and data availability were handled appropriately and comply with open science practices.

 3. Critical Revisions Required for Scientific Soundness

• Clarify bibliometric tools, R packages used, and analysis thresholds in the Methodology section.
• Refine the literature review structure with subheadings and better synthesis of theoretical gaps.
• Reduce citation redundancy and ensure balanced scholarly references in all sections.
• Differentiate clearly between Results and Discussion; interpret figures explicitly within the text.
• Strengthen abstract clarity and specify context behind major trends.
• Conduct comprehensive copy-editing to correct grammatical issues and improve readability.

4. Final Approval Status

 REVISIONS REQUIRED. The article is well-conceived and relevant but requires moderate revisions to be considered scientifically sound. Upon addressing the outlined issues, it will make a valuable contribution to the scholarship on educational digital leadership.

 5. Reviewer Form Questions – Detailed Responses

 1. Is the work clearly and accurately presented and does it cite the current literature?

 Answer: PARTLY

 The manuscript is generally clear and coherent in its purpose and design. However, it occasionally suffers from overly long sentences and a lack of syntactic clarity, especially in the introduction and literature review. The citations are mostly current, but heavily centered around a narrow set of authors (e.g., Karakose, Altinay). This creates an echo chamber that underrepresents broader scholarly discourse. The authors are encouraged to diversify their citations to incorporate geographically and conceptually broader research perspectives and reduce over-reliance on a limited number of sources.

 2. Is the study design appropriate and is the work technically sound?

 Answer: PARTLY

 The use of bibliometric analysis is appropriate for the research questions. However, the methodology section does not detail the specific software packages or algorithms used (e.g., bibliometrix, clustering parameters). The network analysis and thematic evolution are technically valid, but the absence of descriptions for threshold settings or analytical parameters weakens reproducibility. Authors must clarify the tools, settings, and rationale behind analytical choices to ensure technical transparency.

 3. Are sufficient details of methods and analysis provided to allow replication by others?

 Answer: NO

 Although a PRISMA-style diagram and basic inclusion criteria are provided, the exact search strings, field tags, Boolean operators, and parameter thresholds used in RStudio are omitted. This omission significantly limits the replicability of the study. The authors should provide the full search syntax, specify field limitations (title, abstract, keywords), and explain their data cleaning and keyword normalization processes.

 4. If applicable, is the statistical analysis and its interpretation appropriate?

 Answer: PARTLY

 Though traditional statistical testing is not required, bibliometric indicators (e.g., H-index, co-authorship density, citation metrics) require clear interpretation. Some claims—such as thematic maturity or field saturation—lack quantitative justification. The authors are advised to define bibliometric indices more precisely and support trends with visual evidence or model-based explanations.

 5. Are all the source data underlying the results available to ensure full reproducibility?

 Answer: YES

 The dataset has been made openly available on Zenodo with a working DOI. This commendable effort complies with open science principles and allows external validation of the bibliometric analysis. No further action required here.

 6. Are the conclusions drawn adequately supported by the results?

 Answer: PARTLY

 The conclusions summarize the bibliometric trends effectively but occasionally extrapolate beyond the data. For example, assertions about thematic maturity or policy directions lack direct evidence. Authors should temper such claims, clearly separate data-driven inferences from broader interpretations, and explicitly link implications to the specific findings derived from their analysis.

Is the work clearly and accurately presented and does it cite the current literature?

Partly

If applicable, is the statistical analysis and its interpretation appropriate?

Partly

Are all the source data underlying the results available to ensure full reproducibility?

Yes

Is the study design appropriate and is the work technically sound?

Partly

Are the conclusions drawn adequately supported by the results?

Partly

Are sufficient details of methods and analysis provided to allow replication by others?

No

Reviewer Expertise:

Educational Leadership

I confirm that I have read this submission and believe that I have an appropriate level of expertise to state that I do not consider it to be of an acceptable scientific standard, for reasons outlined above.

# Reviewer report: Reviewer response for version 1

**DOI:** 10.5256/f1000research.183690.r399596

**Published:** 2025-08-22

**Authors:** Asem Obied

**Affiliations:** 1 Palestine Technical University-Kadoorie, Ramallah, Palestinian Territory

Lack of Clear Theoretical Framework: While recent sources are integrated, there is limited discussion about the underlying theoretical frameworks guiding the bibliometric analysis or how these frameworks influence the interpretation of research trends. A clearer articulation of the conceptual lens could strengthen the study.

 Insufficient Discussion of Practical Implications: The implications for policymakers and practitioners are broad but lack specificity. More detailed recommendations based on the findings would enhance the paper’s relevance to real-world educational leadership practice.

 No Examination of Research Quality: The paper maps publication trends and social structures but does not evaluate the quality, citation impact, or validity of the publications analyzed, which could influence the interpretation of research influence and innovation .

 Limited Consideration of Emerging Topics: Although thematic evolution is mentioned, the paper might benefit from deeper analysis of emerging topics like AI, data analytics, and leadership 4.0 to highlight future directions more explicitly.

 Language and Clarity: The manuscript could improve clarity in some sections, especially in articulating complex bibliometric methods and their significance. Clearer explanations would make the paper more accessible to a broader audience.

 Temporal Gaps in Data: The drop in publications observed in 2024 is noted but not thoroughly analyzed—possible reasons such as delays in publication, changes in funding, or shifts in research focus are not discussed.

 The pictures in the figures are not clear.

 There is inaccuracy in the years, sometimes the researcher uses the year 1993 and sometimes the year 2010.

 There is no match between Figure 5 and its comment.

Is the work clearly and accurately presented and does it cite the current literature?

Yes

If applicable, is the statistical analysis and its interpretation appropriate?

Yes

Are all the source data underlying the results available to ensure full reproducibility?

Yes

Is the study design appropriate and is the work technically sound?

Yes

Are the conclusions drawn adequately supported by the results?

Yes

Are sufficient details of methods and analysis provided to allow replication by others?

Yes

Reviewer Expertise:

NA

I confirm that I have read this submission and believe that I have an appropriate level of expertise to confirm that it is of an acceptable scientific standard, however I have significant reservations, as outlined above.

## References

[1] : Digital leadership in the academic environment: A systematic literature review. *Social Sciences & Humanities Open* .2025;11: 10.1016/j.ssaho.2025.101542 10.1016/j.ssaho.2025.101542
